# Embryologically based radical hysterectomy as peritoneal mesometrial resection (PMMR) with pelvic and para-aortic lymphadenectomy for loco-regional tumor control in endometrial cancer: first evidence for efficacy

**DOI:** 10.1007/s00404-015-3956-y

**Published:** 2015-11-23

**Authors:** Rainer Kimmig, Antonella Iannaccone, Bahriye Aktas, Paul Buderath, Martin Heubner

**Affiliations:** Department of Gynaecology and Obstetrics, West-German Tumour Center, University Hospital Essen, Hufelandstr. 55, 45147 Essen, Germany

**Keywords:** Endometrial cancer, Peritoneal mesometrial resection, PMMR, Pelvic and para-aortic lymphadenectomy, Embryologically based compartmental surgery, Indocyanine-green, ICG, Robotic surgery

## Abstract

**Objective:**

To evaluate the feasibility and efficacy of embryologically based compartmental surgery for locoregional tumor control in intermediate and high risk endometrial cancer: peritoneal mesometrial resection with therapeutic pelvic and para-aortic lymphadenectomy by robotically assisted laparoscopy.

**Methods:**

75 consecutive surgically treated patients with uterine malignancies have been analyzed. 68 patients with histologically proven endometrial cancer and complete robotically assisted surgery have been included in this study on morbidity and oncological outcome. 56 % of the patients were at intermediate/high risk with either stage IAG3 or IB (*n* = 22) or stage II–IV (*n* = 16). Adjuvant EBRT was offered to three patients only (4 %), whereas five received isolated vaginal brachytherapy (7 %). Indocyanine-green (ICG) fluorescence lymphography is demonstrated being useful for additional intraoperative visualization of the compartment borders and lymphatic drainage to the postponed lymph compartments.

**Results:**

After a mean follow-up of 32 months, there were only two loco-regional recurrences (2.9 %). Both recurrences were apparently cured by salvage therapy. 9 patients died; 6 (8.8 %) from metastatic disease (5) or unknown cause (1), 3 (4.4 %) from intercurrent disease without evidence of disease. One patient (1.4 %) experienced a peritoneal carcinosis and is alive. There were 8/68 perioperative complications (12 %). No perioperative mortality was observed.

**Conclusions:**

Embryologically defined compartmental surgery by robotically assisted laparoscopy seems to be feasible and safe in endometrial cancer. The low loco-regional recurrence rate of 2.9 % in spite of a very low percentage of adjuvant radiotherapy and 56 % of intermediate/high risk tumors should stimulate to initiate a multicentre trial to evaluate the value of compartmental surgery for prevention of locoregional recurrence in endometrial cancer.

## Introduction

Endometrial Cancer develops within the embryologically determined Müllerian compartment. Organ compartments are derived from their developmental precursors and arranged topologically in defined tissue domains—i.e., morphogenetic fields [1]. During tissue growth and differentiation, the compartment boundaries are maintained and well controlled between the organ compartments [2]. The ontogenetic theory suggests clinical cancer as the result of pathological reactivation of normally blocked developmental programs during embryogenesis in retrograde order [3]; tumor progression is thus confined for a very long time to the mature tissue embryologically derived from the same organ compartment as could be demonstrated for cervical cancer [4]. This is also true for the spread to the corresponding draining lymphatic system of the compartment which is developed by sprouting from embryonal veins [5]; it is connected to the embryologically derived lymphatic compartments along the main pelvic and aortic vessels via so called “intercalated” lymph nodes. Since regional draining lymph nodes contain the same topic information as the drained organ compartment, this lymph compartment is also permissive for growth since tumor cells are identified to be intrinsic [3].

As a consequence of these findings, it should be possible to remove a malignant tumor and its “loco-regional continuous and lymphatic spread” by removing the compartments at risk entirely as long as it is in ontogenetic stage I according to [3]. In case of endometrial cancer this would correspond to the Müllerian compartment together with the regionally draining lymph compartments and the intercalating lymph nodes. Ideally, there should be no risk of recurrence due to local or lymphatic spread in the pelvis and para-aortic region without any additional treatment. Radiotherapy could thus be avoided completely in primary treatment.

However, if this would hold true, there may still be several reasons for loco-regional recurrence:

*First* Although being part of Müllerian compartment, the vagina has to be preserved for functional reasons—enhancing the risk for intra-compartmental recurrence. This risk may be reduced by wide resection and potentially application of postoperative local irradiation by vaginal brachytherapy.

*Second* Inability to completely remove involved Müllerian compartment or the regional lymph compartments at risk, particularly if nodes are involved.

*Third* There may be surgical tumor contamination by spilling tumor tissue to the wounds, which normally contain remnants of Müllerian compartment, such as vagina and mesocolpia; it seems that this risk rises with dedifferentiation of the tumor and involvement of the lymphatic system as has been shown by Mariani et al. for vaginal recurrence [6]. The risk of direct contamination, at least, may be reduced by prevention of intraoperative spilling of vital cells by closing fallopian tubes and the cervical channel prior to the surgery (e.g., coagulation, alcohol sponge).

In contrast to the above-mentioned factors causing loco-regional recurrence, distant recurrence cannot be prevented by surgery. However, there is some evidence that adjuvant chemotherapy may reduce the risk of blood borne metastases; this leads presently to a shift of paradigm in primary endometrial cancer treatment replacing adjuvant radiation more and more by adjuvant chemotherapy in intermediate/high risk situations [7, 8].

Basic findings with respect to embryonic development of organ compartments, tissue boundary control and interaction with tumor progression and tumor spread as outlined already enabled to develop a new concept of compartmental surgery in endometrial cancer. Initiated by M. Höckel the “Peritoneal MesoMetrial Resection (PMMR)” as a distinct type of radical hysterectomy has been defined and combined with the pelvic and para-aortic therapeutic LymphadeNEctomy (tLNE). Principles and technique have been recently described in detail [9, 10].

In the present paper data of the first 75 patients operated on malignancies of uterine corpus by robotic surgery are reported. Clinical results and follow-up of 68 consecutive patients with endometrial cancer stage I–IV were evaluated. Additionally intraoperative visualization of the Müllerian compartment and the draining lymph vessels to the postponed lymph compartments by indocyanine-green fluorescence [11] is demonstrated for the organ (sub) compartment, the intercalated region and the regional lymph compartments.

## Materials and methods

### Patient characteristics

Total cohort consisted of 75 consecutively treated patients with malignancy of uterine corpus. For clinical perioperative analysis and follow-up the following inclusion criteria were defined: endometrial cancer including carcinosarcoma, hysterectomy and/or therapeutic lymphadenectomy performed by robotic surgery (da Vinci Si, Intuitive Surgical Inc.^®^) and no distant metastases (excl. peritoneal carcinosis) at primary treatment.

According to these criteria five patients had to be excluded primarily from the analysis: four due to the diagnosis of pure sarcoma (two undifferentiated endometrial sarcomas, one leiomyosarcoma and one embryonal rhabdomyosarcoma), one patient due to primary pulmonary metastases. Due to inclusion criteria, another two of the patients who has been included in complication analysis had to be excluded from further analysis due to early switch to open surgery not receiving robotic surgery (one injury of caval vein and one due to technical difficulties to withhold the bowel not allowing to perform surgery in the required quality).

Thus, the analysis has been performed on the remaining 68 patients. Mean age was 57.7 years ± 11.8. Histology resulted in 60 endometroid, five serous, one clear-cell, one adeno-squamous carcinomas and one carcinosarcoma. FIGO Stage and Grading were determined as follows: IA G1 = 11, IA G2 = 19, IA G3 = 5, IB G1 = 1, IB G2 = 11, IB G3 = 5, II G1 = 1, II G2 = 3, II G3 = 3,IIIA G3 = 1, IIIC G2 = 4, IIC G3 = 3 and IVBG3 = 1. This corresponds to 30 patients (44 %) with low risk (IA, G1 and G2) and 38 patients (56 %) with intermediate/high risk (G3 or IB–IV) tumors.

### Type of surgery

There were different surgical treatments. The surgical strategy based on preoperative findings and patients gave informed consent prior to surgery. (1) in undoubtedly low risk situations: extrafascial simple hysterectomy with BSO. (2) In unclear situations: PMMR and BSO without lymphadenectomy. (3) In intermediate/high risk situation: PMMR, with pelvic ± para-aortic LNE ± omentectomy. The corresponding numbers were: extrafascial hysterectomy with BSO (*n* = 8); radical hysterectomy (PMMR) with BSO (*n* = 20); radical hysterectomy (PMMR) with pelvic ± para-aortic LNE ± omentectomy (*n* = 36). In addition, there were four patients with simple hysterectomy referred to our center for surgical completion: pelvic and para-aortic LNE ± omentectomy ± mesometrectomy depending on the type of prior hysterectomy. The patients were classified, preoperatively, being low risk with G1 Tumors and suggested pT1a, unclear with G2 or suggested depth of infiltration approximating 50 %, and intermediate/high risk all patients with G3, suggested infiltration >50 % and all stages higher than FIGO I. Patients with low risk received extrafascial hysterectomy with BSO, unclear PMMR with BSO and intermediate/high risk PMMR with therapeutic pelvic and para-aortic LNE.

### Type of adjuvant treatment

Three patients (4.4 %) received external beam irradiation (FIGO IA, G3; IB, G2 and IVB. G3), five patients (7.4 %) local brachytherapy (FIGO IB, G2; 3 × FIGO IB, G3 and FIGO II, G3) and 60 patients (88.2 %) no irradiation at all. Thus, in fact 11.8 % were treated with adjuvant radiation therapy instead of 56 % of the patients which should have been treated according ASTRO/ASCO Guidelines [11] with respect to prevention of loco-regional recurrence; all patients were informed about benefit and harm of adjuvant radiotherapy and made their individual decision.

Fourteen patients (20.6 %) received platinum-based chemotherapy, three of them combined with irradiation (two EBR, one BT).

### Observation time

Mean postoperative observation time was 32 months (median 30 months).

### Visualization of compartments by indocyanine-green (ICG) fluorescence

For visualization of the Müllerian and the postponed lymphatic compartments prior to the surgery in total 1.2 ml of indocyanine-green solution at 1.25 mg/ml solution (ICG-Pulsion^®^) has been injected divided in 4 × 0.3 ml portions in some patients. The injection was given into the uterine corpus bilaterally mid corporal 1 cm above the isthmus and at the fundus at 3 and 9 o’clock (5–10 mm depth) using an Iowa trumpet for application (as used for pudendal anesthesia in obstetrics). The application of ICG was performed approximately 15–20 min before docking of the robotic device. All patient were informed about “off label use” of ICG and gave informed consent. Near infrared excitation was used and fluorescence was detected by an appropriate camera system (Intuitive Surgical Inc.) for intermittent intraoperative detection. Following application of the dye a Hohl-cup (Storz^®^) has been fixed to the cervix by a suture closing the cervical channel with an alcohol sponge to prevent spilling of vital cells. In addition fallopian tubes have been coagulated at the beginning of the surgery.

### Statistical analysis

Descriptive analyses are done, only. Values are given in absolute numbers or percentage.

## Results

### Compartment visualization by indocyanine-green application to the uterine corpus

Although compartment visualization by ICG-fluorescence will be described in detail elsewhere, a short introduction into the findings will be given with respect to endometrial cancer. This may serve as an intraoperative orientation with respect to the technique of PMMR for surgery in endometrial cancer described earlier [9]. Due to the dense, lymphatic vessel network it is possible to visualize not only the lymph nodes by introducing a marker to the lymphatic system, but also the whole organ compartment and its connections to the draining lymph compartments. Following application of ICG to the mid corporal and fundal myometrium (see “Materials and methods”) the Müllerian sub-compartment of uterine corpus may be visualized.

**Fig. 1:**
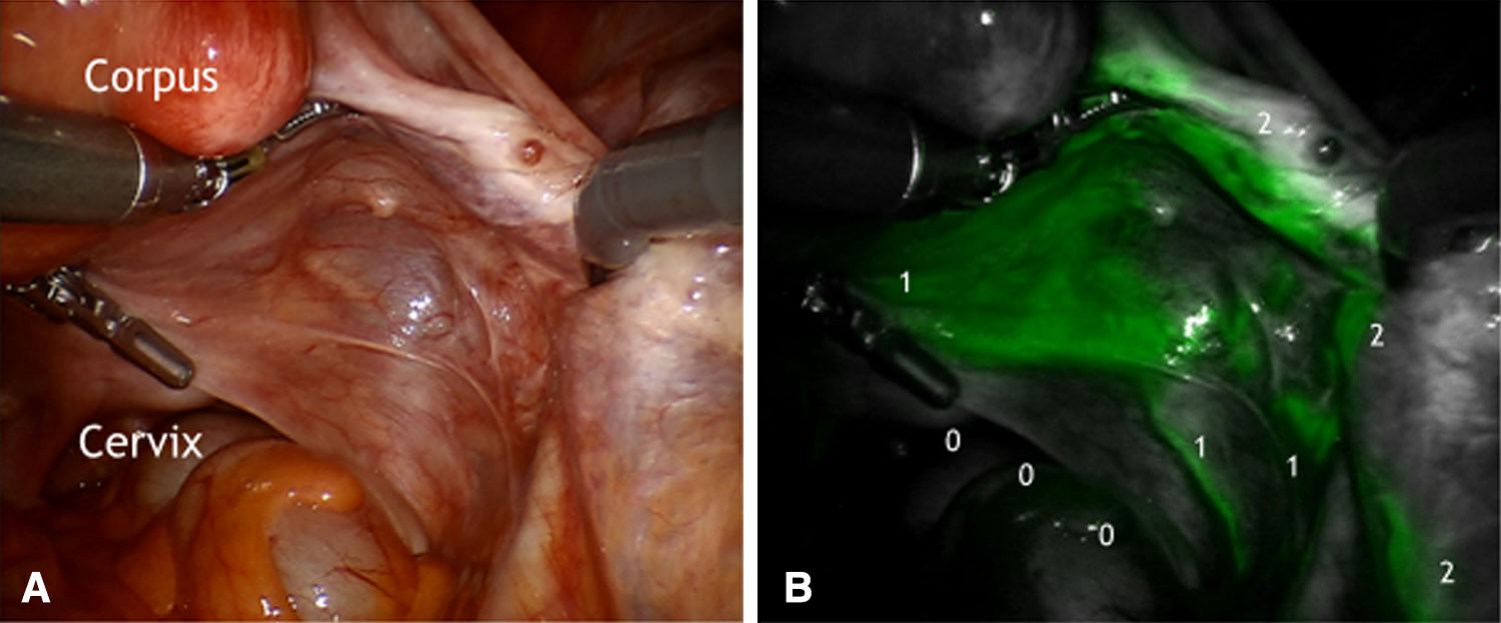
**a** Right posterior aspect of mesometrial and mesonephric compartments. **b** ICG fluorescence along the Müllerian lymphatic mesometrial network and collecting lymph vessels of vascular mesometrium draining to pelvic (111) and paraaortic nodes (222). No fluorescence was found along ligamentous mesometrium (000)

**Fig. 2:**
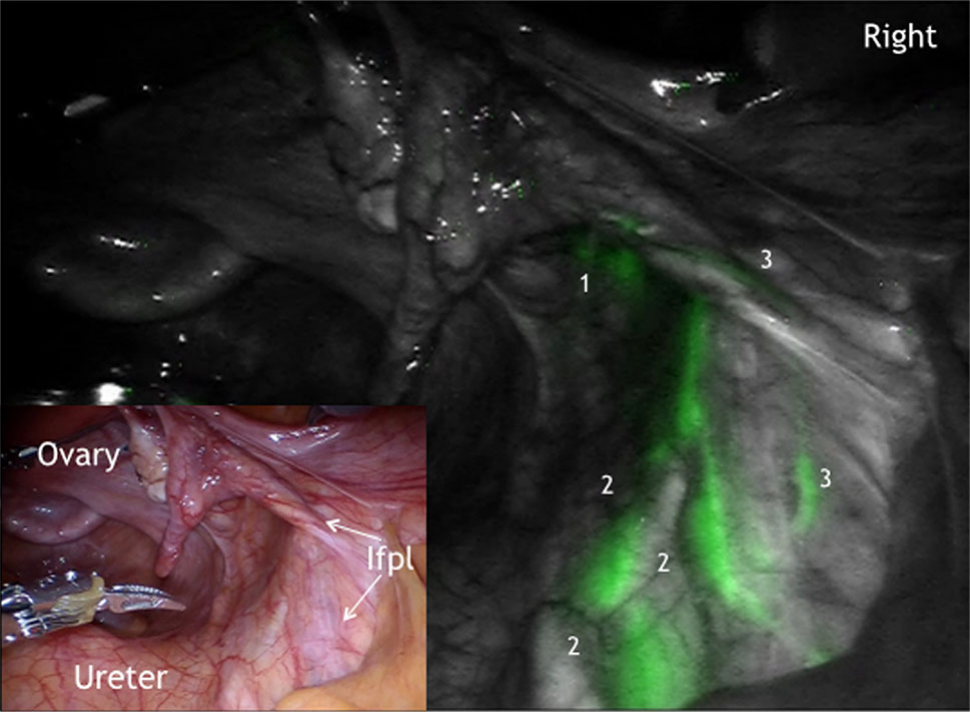
ICG fluorescence in the right paravisceral (1) and common iliac lymph basin (222) and the mesonephric connecting lymph channels along infundibulopelvic ligament (Ifpl, 33)

**Fig. 3:**
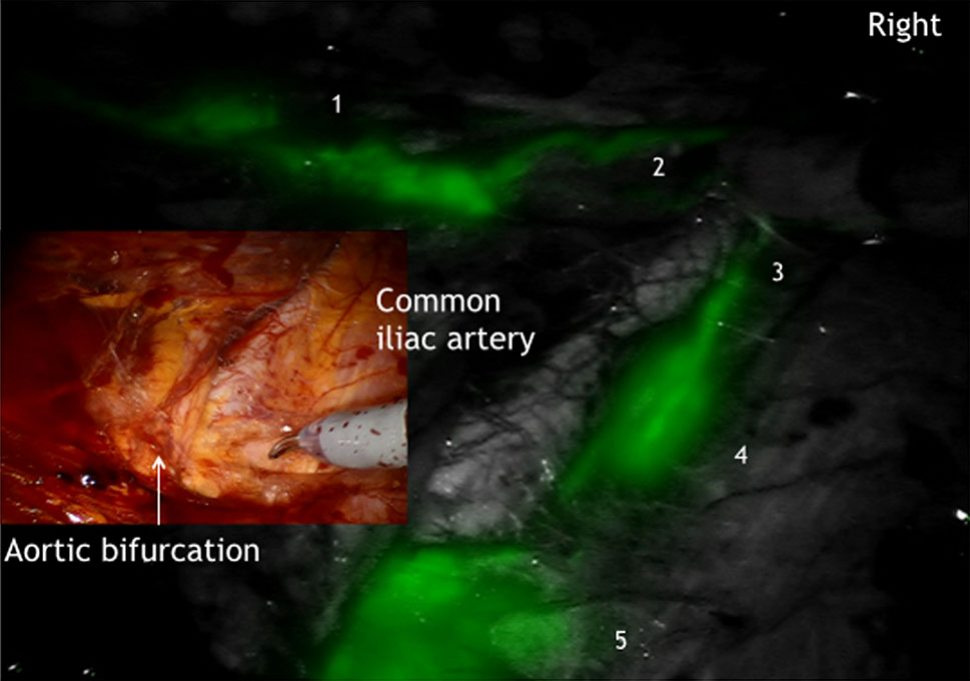
Lymphatic drainage from Müllerian compartment (*1*) via paravisceral (*2*), external (*3*) and common iliac (*4*) to mesenteric para-aortic (*5*) lymph compartment on the right

**Fig. 4:**
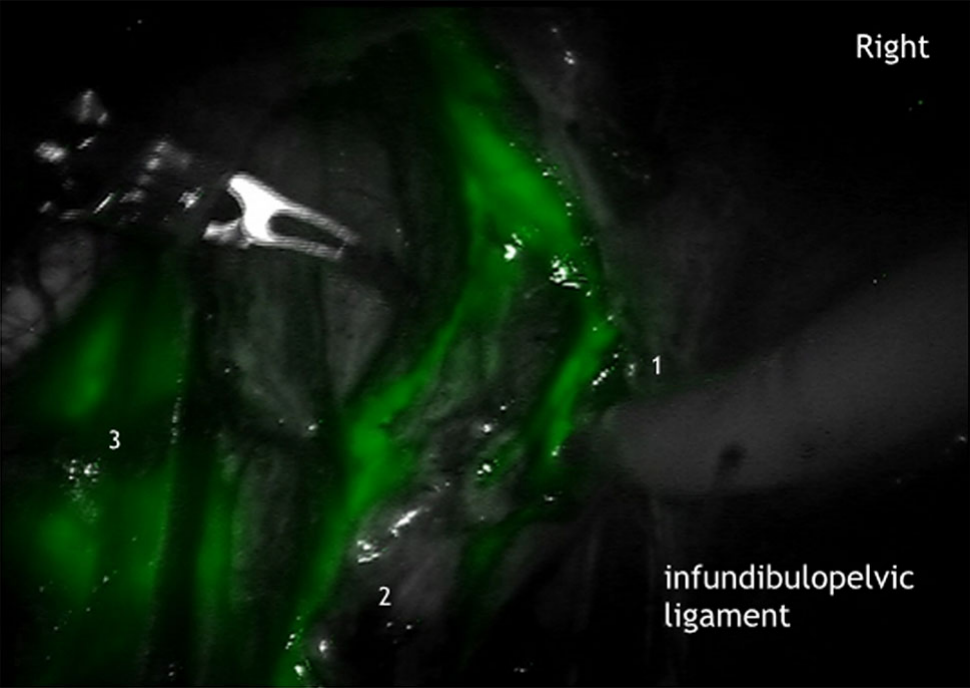
Mesonephric lymphatic truncs along ovarian vein (*1*) and artery (*2*) and common iliac lymph basin (*3*) on the right

**Fig. 5:**
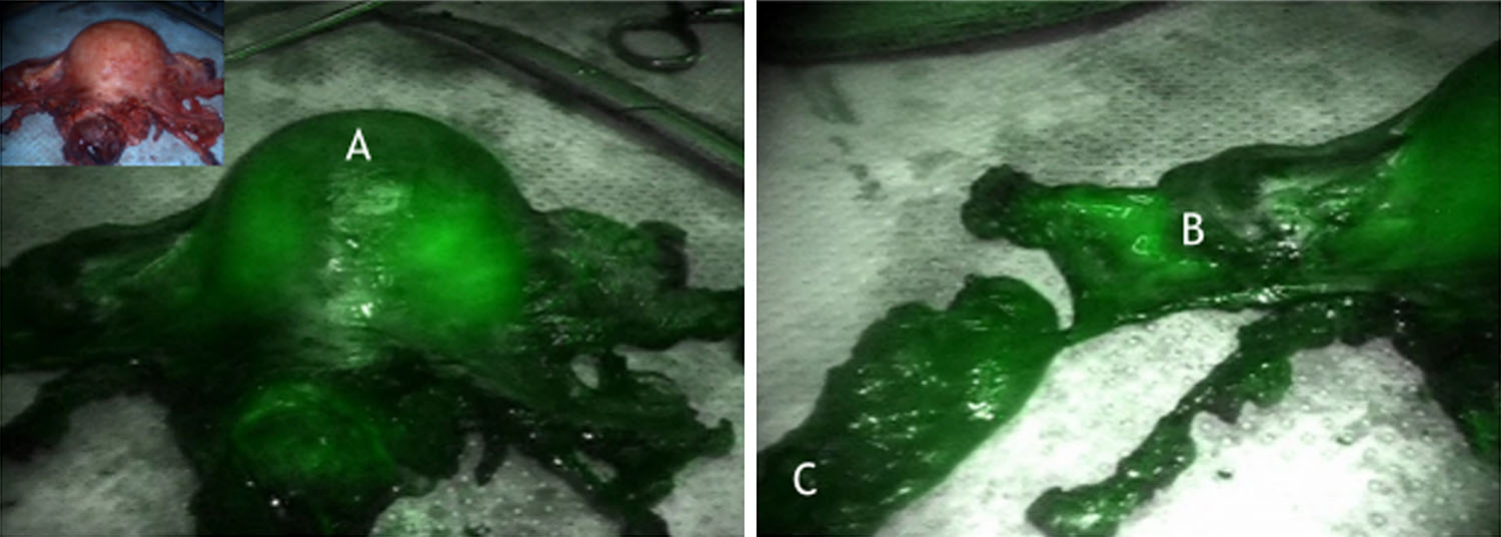
ICG fluorescence of the corporal Müllerian compartment (*A*) and the vascular mesometrial utero-ovarian lymphatic network (*B*) and paravisceral lymph basin (*C*) in the final specimen

It could be demonstrated that there is an ipsilateral drainage from midline to lateral. As suggested intraoperative lymphography of the corporal uterine Müllerian sub-compartment by ICG showed two pathways for the transport of fluorescent lymphatic fluid: first, along the uterine vessels passing the vascular mesometrium—mainly supra-ureteral—to the pelvic nodes along external and internal iliac vessels and second, along the ovarian, mesonephric pathway to the para-aortic nodes. On the other hand, there was no drainage at all along the ligamentous mesometrium (i.e. “sacro-uterine ligament) as it would be typical for drainage of the cervical sub-compartment (Fig. 1). It seems that regular lymphatic drainage from the corporal sub-compartment follows predominantly the ventral pathways to the proximal para-visceral, proximal external, common iliac lymph basins (Fig. 2) and the mesenteric para-aortic lymph basins (Fig. 3). The mesonephric drainage along the ovarian vessels to the higher para-aortic nodes may also be followed along the corresponding lymph vessels (Fig. 4). The fluorescence persists within the Müllerian compartment during hours so it can be still visualized in the removed specimen (Fig. 5).

### Patient outcome following compartmental surgery or endometrial cancer

In total, 70 patients were treated consecutively for endometrial cancer. Two patients had to be converted to open due to an injury of the caval vein (1) and inability to perform adequate surgery (1) (2 = 2.9 %). Stage, patient characteristics and treatment procedures of the remaining 68 patients receiving the complete robotic procedure are summarized in “Materials and methods” for analysis of the clinical course.

### Complications

In total, we observed 10/70 (14.2 %) and 8/68 (11.8 %) complications, respectively. There was one intraoperative injury (1.5 %) of the small bowel in a patient with lymphadenectomy only following extrafascial hysterectomy/BSO, which required suture of the lesion and secondarily resection of a small bowel segment without further sequelae. Postoperatively, there were three complicated lymphoceles, one mild and transient bladder dysfunction, one deep venous thrombosis of right lower limb, one superficial venous thrombosis of Vena saphena parva and one transitory ischaemic attack (*n* = 7). No perioperative mortality was observed.

There were no transfusions but one in a patient with preoperative hemoglobin level of 8.7 g/dl.

All complications, but one, occurred in patients who received a lymphadenectomy as part of the surgery. The transient bladder dysfunction was observed in a patient with PMMR/BSO without lymph node dissection. Grade I complaints of bladder function persisted for about 1 year and disappeared thereafter completely without medication. Comparing simple hysterectomy/BSO (*n* = 8) with PMMR/BSO (*n* = 20), no other complications occurred in both groups. No transfusion was required. Mean difference (±SD) of pre- and postoperative hemoglobin was 1.6 ± 0.9 and 2.2 ± 1.0 g/dl, respectively. There was no difference in length of hospital stay. Thus, morbidity of PMMR/BSO without LNE has been comparably low as for simple hysterectomy/BSO.

### Oncological outcome

As outlined in Materials and Methods there were 38/68 patients intermediate/high risk situations (56 %) facing a higher probability for recurrence. Three patients only received adjuvant external beam irradiation (4.4 %) and 5 (7.4 %) received local vaginal afterloading, whereas 14 (20.6 %) received adjuvant chemotherapy.

After a mean follow-up of 32 months, 8 recurrences were observed (11.8 %):

1. There were in total two loco-regional recurrences (2.9 %), 27 and 25 months following primary treatment. The two patients had not received any adjuvant irradiation.

First, a 71-year-old patient had been diagnosed with FIGO stage IIIc—pT1b, G2, pN1 (3/46, two pelvic and one para-aortic node) and had been treated by PMMR/BSO and pelvic and secondary para-aortic lymphadenectomy; she received carboplatin/liposomal doxorubicin adjuvant chemotherapy and no irradiation; she developed an isolated recurrence in the right common iliac region and had been treated by salvage surgery and postoperative external irradiation; she is presently free of recurrence at 47 months.

The second 63-year-old patient FIGO stage Ib—pT1b, pN0, G2 received no adjuvant treatment. She developed a carpet-like recurrence of the upper two-thirds of the vagina (Müllerian compartment) and has been irradiated. Due to persistent viable tumor, secondarily robotic radical colpectomy has been performed. At present, this patient is also free of recurrence (OT 48 months).

2. Six patients (8.8 %) developed distant metastases of the liver, lungs, bone, ± adrenal gland or peritoneal carcinosis (5) or peritoneal carcinosis only (1). All patients died from disease between 6 and 26 months after primary treatment except for the patient with peritoneal carcinosis who is presently alive with disease. 4/6 (67 %) patients had received radiotherapy and/or chemotherapy.

Patient 1, 78 years (18 months): FIGO IIIC, pT3b, pN1 24/46, G2, L1, Rx; treatment PMMR, BSO, pelvic and para-aortic LNE, adjuvant carboplatin/paclitaxel chemotherapy.

Patient 2, 41 years (6 months): FIGO IIIC, pT3b, pN1 5/67, G3, L0, R0; treatment PMMR, BSO, pelvic and para-aortic LNE, adjuvant carboplatin/paclitaxel chemotherapy.

Patient 3, 63 years (26 months): FIGO II, pT2, pN0 0/42, G3, L0, R0, serous carcinoma; treatment LAVH e.m., PMMR completion, pelvic and para-aortic LNE, omentectomy, adjuvant carboplatin/paclitaxel chemotherapy and vaginal afterloading.

Patient 4, 27 years (17 months): FIGO Ia, pT1a, pN0 0/28, G3, L0, R0; treatment PMMR and pelvic LNE only, adjuvant external beam irradiation and vaginal brachytherapy. Additional remark: BMI 55, preoperative hemoglobin 8.7 g/dl; the only patient who required transfusion.

Patient 5, 78 years (6 months): FIGO Ib, pT1b, pN0 0/40, G3, L0, R0; e.m. hysterectomy and BSO; robotic pelvic and paraaortic LNE, adjuvant carboplatin/paclitaxel chemotherapy, vaginal afterloading.

Patient 6, 62 years (25 months): pT1a, pNx, GIII, serous; no adjuvant treatment (refused); she developed isolated malignant ascites due to peritoneal carcinomatosis; she received platinum-based chemotherapy and is alive.

There were another three patients who died without evidence of disease [cerebral vascular insufficiency, dementia and diabetes (73 years, 25 months p.s.) and renal failure (76 years; 24 months p.s.) and secondary esophageal cancer (86 years, 43 months p.s.) and one patient with unclear situation (82 years; 9 months p.s.)]. In summary, 6/68 patients died suffering from distant tumor progression or unknown cause according to 8.8 %.

Thus, at mean follow-up at 32 months after surgical treatment 59/68 (86.8 %) patients are alive and 58/68 (85.3 %) without evidence of disease although 56 % of the patients suffered from intermediate/high risk tumors.

## Discussion

Outcome following treatment of endometrial cancer is excellent for about 60 % of patients with low risk disease. However, in intermediate and high risk situation there is substantial risk for local, regional and distant recurrence and death of disease especially if there is involvement of lymph nodes or peritoneal carcinosis [12]. Primary radiotherapy of endometrial cancer is less effective than surgery and adjuvant radiotherapy does not enhance survival [8, 13]; in addition, long-term survival following adjuvant radiotherapy shows significant morbidity with respect to the bowel and bladder function, the sexuality and is accompanied by an nearly twofold increase of incidence of secondary malignancies [12, 14, 15]. Thus, consequently, surgery ideally has to be performed in a way not requiring additional adjuvant radiotherapy with respect to control for local and regional disease. Certainly, this may still have no impact on control of distant disease. However, chemotherapy may reduce risk of distant recurrence [7, 8] and may be indicated in intermediate/high risk situations.

With respect to the concept of ontogenetic tumor development the compartment of risk for endometrial cancer growth and recurrence has to be defined. In low risk tumors this might be the Müllerian sub-compartment of tumor origin, followed by the Müllerian compartment in conjunction with the draining Müllerian pelvic and mesonephric para-aortic lymph compartments with increasing dedifferentiation and regression of embryonic development. With further embryonic regression the coelomic compartment especially in type II endometrial cancer and the vesico-urinary system may follow. Finally, in high ontogenetic stage the tumor may be capable to form distant metastases in different organ systems [3].

Thus, to achieve loco-regional control in endometrial cancer of intermediate/high risk it is necessary to remove the Müllerian compartment together with the loco-regional lymphatic compartments of risk (i.e., with the same topic information as the Müllerian compartment). The resulting surgical procedure based on this concept has been already described [9, 10].

In intermediate/high risk endometrial cancer treated by conventional hysterectomy and even systematic lymphadenectomy there is still a high local and regional recurrence rate even in experienced centers for gynaecologic oncology [16]. This is true although consequent adjuvant treatment with radiotherapy has been added for loco-regional control with or without chemotherapy. The authors reported recurrences in 22/128 patients (17 %) and death of 20/128 patients (15 %) after a median follow up of 30 months. The corresponding figures in our study were 11.8 and 8.8 %, respectively, with 32 months of median follow up. Taking into account that only 56 % of our patients suffered from intermediate/high risk disease and none of the low risk patients experienced a recurrence, the numbers correspond very well. Also with respect to the number of positive nodes, there were 12 % in the total cohort and 21 % positive nodes when corrected for intermediate/high risk situations (equal to [16]). However, in contrast to this study our patients have not been routinely treated by adjuvant radiotherapy (88 % without any irradiation) and did not experience a single recurrence of the upper vaginal vault compared to seven patients (5.5 %) in the cited study. In total, there were two regional recurrences (2.9 %) which could both be cured by salvage therapy. Thus, there was a trend to lower locoregional recurrences in our study even when corrected for the high risk patients, i.e., 5 vs. 10 % (13/128) in [16]. Our results contrast also to large studies which also report a loco-regional recurrence rate up to 15 % in intermediate/high risk situation with adjuvant treatment and assume 20–30 % vaginal recurrences without adjuvant treatment in the high risk situation [17]; they were also lower than expected calculating individually estimated recurrence rates using the nomogram derived from the PORTEC I and II data for stage 1 disease [18].

In summary, we could show that compartmental surgery in patients with endometrial cancer is feasible and safe. There is no evidence for an increase in complications and low perioperative morbidity. In particular—except for one transient mild bladder dysfunction—no relevant difference in morbidity could be found comparing PMMR/BSO with simple hysterectomy/BSO if not combined with lymphadenectomy in this study.

The number of patients treated in this study is still too low to draw definitive conclusions with respect to oncological outcome: however, it seems to confirm the expectation that embryologically compartment-based oncologic surgery respecting the concept of ontogenetically determined tumor progression might reduce the risk of loco-regional recurrence in endometrial cancer by surgery alone without additional adjuvant radiotherapy; these findings seem to be comparable to the data in cervical (TMMR [19]) and rectal (TME [20]) cancer.

Although these data should be interpreted with caution, they may justify to evaluate this concept in endometrial cancer in a multicentric approach concerning loco-regional control, whereas distant control has to be investigated independently with respect to adjuvant chemotherapy.
